# Inappropriate activation of the renin-angiotensin system improves cardiac tolerance to ischemia/reperfusion injury in rats with late angiotensin II-dependent hypertension

**DOI:** 10.3389/fphys.2023.1151308

**Published:** 2023-06-14

**Authors:** Zuzana Husková, Soňa Kikerlová, Matúš Miklovič, Petr Kala, František Papoušek, Jan Neckář

**Affiliations:** ^1^ Center of Experimental Medicine, Institute for Clinical and Experimental Medicine, Prague, Czechia; ^2^ Department of Pathophysiology, 2nd Faculty of Medicine, Charles University, Prague, Czechia; ^3^ Department of Cardiology, 2nd Medical Faculty, Charles University and University Hospital Motol, Prague, Czechia; ^4^ Laboratory of Developmental Cardiology, Institute of Physiology, Academy of Sciences of the Czech Republic (ASCR), Prague, Czechia

**Keywords:** renin-angiotensin system, ischemia/reperfusion injury, ANG II-dependent hypertension, AT1 receptor antagonist, P-V analysis

## Abstract

The aim of the study was to clarify the role of the interplay between hypertension and the renin-angiotensin system (RAS) in the pathophysiology of myocardial ischemia/reperfusion (I/R) injury. We hypothesized that in the late phase of hypertension with already developed signs of end-organ damage, inappropriate RAS activation could impair cardiac tolerance to I/R injury. Experiments were performed in male *Cyp1a1-Ren-2* transgenic rats with inducible hypertension. The early phase of ANG II-dependent hypertension was induced by 5 days and the late phase by the 13 days dietary indole-3-carbinol (I3C) administration. Noninduced rats served as controls. Echocardiography and pressure-volume analysis were performed, angiotensins’ levels were measured and cardiac tolerance to ischemia/reperfusion injury was studied. The infarct size was significantly reduced (by 50%) in 13 days I3C-induced hypertensive rats with marked cardiac hypertrophy, this reduction was abolished by losartan treatment. In the late phase of hypertension there are indications of a failing heart, mainly in reduced preload recruitable stroke work (PRSW), but only nonsignificant trends in worsening of some other parameters, showing that the myocardium is in a compensated phase. The influence of the RAS depends on the balance between the vasoconstrictive and the opposed vasodilatory axis. In the initial stage of hypertension, the vasodilatory axis of the RAS prevails, and with the development of hypertension the vasoconstrictive axis of the RAS becomes stronger. We observed a clear effect of AT1 receptor blockade on maximum pressure in left ventricle, cardiac hypertrophy and ANG II levels. In conclusion, we confirmed improved cardiac tolerance to I/R injury in hypertensive hypertrophied rats and showed that, in the late phase of hypertension, the myocardium is in a compensated phase.

## 1 Introduction

Twenty-five years after its designation as an emerging epidemic, heart failure (HF) remains a major clinical and public health problem. The lifetime risk of HF remains high, with variation across racial and ethnic groups ranging from 20% to 45% after 45 years of age (Tsao et al., 2022). The incidence of HF with preserved/normal ejection fraction is increasing and, in contrast, the incidence of HF with reduced ejection fraction is decreasing, whereas both HF subtypes have similar all-cause mortality rates. The main risk factors for HF are high blood pressure (HBP), myocardial hypertrophy and ischemia (Elgendy et al., 2019). From 2009 to 2019, the death rate caused by HBP increased 34.2%, and the actual number of deaths related to HBP rose up to 65.3% (Tsao et al., 2022). Nearly 70% of all HF syndromes can be attributed to underlying ischemic heart disease (IHD) (Elgendy et al., 2019). According to a multiethnic population study, the prevalence of unexplained left ventricular hypertrophy (LVH) in the society is estimated to be 0.2% even up to 1.4% (Massera et al., 2019).

The inappropriate activation of the renin-angiotensin system (RAS) is a crucial mediator of HF development and progression, moreover the vasodilatory axis of the RAS is a key regulator in myocardial remodeling and development of heart failure (Wang et al., 2017). Angiotensin 1–7 (ANG 1–7), which is hydrolysed from angiotensin II (ANG II) by angiotensin-converting enzyme 2 (ACE2), exhibits potent antihypertrophic, antifibrotic, antioxidant and vasoprotective actions to counteract the harmful effects of ANG II signaling with promising clinical significance in the settings of both HF with reduced and preserved ejection fraction (Patel et al., 2016; Yu et al., 2018). Elevated ANG 1–7/ANG II ratio was identified as an independent and incremental predictor of beneficial outcomes, higher survival rate, and decreased hospitalization duration (Binder et al., 2019; Oudit et al., 2020).

Two decades ago, new strain of inbred transgenic rats with inducible hypertension [strain name, TGR (*Cyp1a1–Ren-2*)] was generated using the cytochrome P-450 promoter, *Cyp1a1*, to regulate the expression of the mouse *Ren-2* renin gene (Kantachuvesiri et al., 2001). After exposure to natural xenobiotics such as indol-3-carbinol (I3C), the renin transgene is rapidly expressed primarily in the liver, leading to increased ANG II levels and the development of ANG II-dependent form of hypertension. We and others have shown that gene expression, endogenously produced ANG II levels, and the degree of subsequent hypertension can be precisely controlled in a dose- and time-dependent manner as well as in a reversible manner (Peters et al., 2008, 2009, 2012; Erbanová et al., 2009; Husková et al., 2010, 2016, 2021; Honetschlägerová et al., 2011b; Cunningham et al., 2013; Sporková et al., 2014; Jíchová et al., 2016; Sedláková et al., 2018). We chose this model as the optimal model for studying the relationship between the activity of endogenous RAS, the phase of hypertension and the degree of cardiac ischemic tolerance.

In the current study, we decided to clarify the role of the interplay between hypertension and RAS in the pathophysiology of myocardial ischemia-reperfusion (I/R) injury. In our recent study we suggested moderately increased cardiac ischemic tolerance to I/R insult (Husková et al., 2021). However, we showed that activation of AT1 receptors by locally produced ANG II in the heart is not the mechanism underlying infarct size reduction in the early stage of hypertension. If hypertension plays a dual role in myocardial I/R injury (Červenka et al., 2018), it can be assumed that in the late phase of hypertension with already developed signs of end-organ damage, inappropriate RAS activation impairs cardiac tolerance to I/R injury. Thus, we evaluated changes in cardiac function/structure in late phase of hypertension following I/R injury in presence or absence of angiotensin II receptor antagonist. To further elucidate the underlying mechanism (s), we evaluated cardiac structure and function using non-invasive echocardiography (ECHO) and invasive pressure-volume (P-V) analysis of the left ventricle (LV).

## 2 Materials and methods

### 2.1 Ethical approval, animals, and chemicals

The animal experiments were approved by the Animal Care and Use Committee of the Institute for Clinical and Experimental Medicine (IKEM), Prague, consequently, by the Ministry of Health of the Czech Republic (project decision MZDR 32747/2017-3/OVZ), in accordance with the guidelines and practices established by the European Convention on Animal Protection and Guidelines on Research Animal Use, as described in the Directive 2010/63/EU as amended by Regulation (EU) 2019/1010 (http://data.europa.eu/eli/dir/2010/63/2019-06-26).

Experiments were performed in male *Cyp1a1-Ren-2* transgenic rats, at initial age of 3 months and weight of 300 g. In this inbred strain of transgenic rats the degree of ANG II-dependent hypertension, gene expression and the level of endogenously produced ANG II can be precisely controlled in a dose- and time-dependent way (Husková et al., 2010; 2016; 2021; Honetschlägerová et al., 2011a; Sporková et al., 2014; Jíchová et al., 2016). The animals were bred and housed at the Center of Experimental Medicine, IKEM, from stock animals supplied by Professor Mullins from the Center for Cardiovascular Science, University of Edinburgh, United Kingdom. Animals were housed under standard conditions (temperature 22°C ± 1°C; relative humidity 40%, 12-h light/dark cycle), fed standard pellet rat chow or rat chow containing 0.3% I3C, and given tap water *ad libitum* or losartan in drinking water. The rats were fasted the day before the start of I3C-induction to ensure they began to eat immediately after rat chow with I3C was given.

*Losartan* (Lozap, Zentiva a. s., Hlohovec, Slovak Republic), specific AT1 receptor antagonist, was given in drinking water at the concentration 100 mg/L, starting 24 h before the onset of I3C-induction to ensure complete blockade of AT1 receptors, and continuing throughout I3C-induction.

### 2.2 Experimental design–series 1

The aim of this series was to evaluate the cardiac morphology and function. The measurements were performed in noninduced rats and the rats after 5 days (5 days) or 13 days (13 days) of I3C-induction, losartan treated or untreated. The following experimental groups were examined:

1. Noninduced rats/untreated (*n* = 9)
2. Noninduced rats/losartan (*n* = 9)
3. 5 days I3C-induced/untreated (*n* = 8)
4. 5 days I3C-induced/losartan (*n* = 8)
5. 13 days I3C-induced/untreated (*n* = 9)
6. 13 days I3C-induced/losartan (*n* = 9)

At the end of experiments, anesthetized rats (Thiopental VAUB, 50 mg/kg i. p., VAUB Pharma a. s., Roztoky, Czech Republic) were intubated with a plastic cannula, relaxed with pancuronium (Pavulon, 0,16 mg/kg, N.V. Orga-non, Oss, Netherlands), and artificially ventilated (rodent ventilator; Ugo Basile, Gemonio VA, Italy, FiO2 = 21%). LV function was invasively assessed by 2 F Pressure-Volume (P–V) micromanometer catheter (Millar Instruments, Houston, TX, United States) introduced into the LV cavity via the right carotid artery as described in our previous studies (Kala et al., 2020, 2021, 2023; Havlenova et al., 2021). To measure the dynamic systolic and diastolic parameters of cardiac functions, the preload was regulated by a 4 F Fogarty catheter (Edwards Lifesciences, Irvine, CA, United States) in the vena cava under the diaphragm introduced via the right jugular vein. The volume signal was calibrated by a blood cuvette calibration system dedicated by the catheter producer (P/N 910-1048, Millar Instruments, Houston, TX, United States). Data were acquired using an 8-channel Power Lab recorder and were analyzed by LabChart PRO software (ADInstruments, Bella Vista, NSW, Australia).

### 2.3 Experimental design–series 2

The aim of series 2 was to determine the level of RAS activity. The following experimental groups were evaluated:

1. Noninduced rats/untreated (*n* = 8)
2. Noninduced rats/losartan (*n* = 11)
3. 5 days I3C-induced/untreated (*n* = 11)
4. 5 days I3C-induced/losartan (*n* = 9)
5. 13 days I3C-induced/untreated (*n* = 11)
6. 13 days I3C-induced/losartan (*n* = 8)

At the end of experiments, all rats were decapitated to collect blood and tissue samples for analysis. The left ventricular weight (LVW) to tibia length ratio was used to evaluate the degree of left ventricle hypertrophy. Based on our and others’ experiences (Honetschlägerová et al., 2011a; Sporková et al., 2014; Husková et al., 2016; Jíchová et al., 2016), this is the most appropriate index to assess cardiac hypertrophy when the development of hypertension in untreated I3C-induced rats is associated with profound body weight (BW) loss.

Plasma and tissue ANG II and ANG 1–7 levels were measured by competitive radioimmunoassay (RIA) using the commercially available (RB320, DIAsource, Louvain-la-Neuve, Belgium) and custom-made (Immunotech s. r.o., Prague, Czech Republic) RIA kits, respectively. This approach developed by Navar et al. (1994) has been modified, verified and is routinely used in our laboratory (Husková et al., 2006, 2010, 2016). Briefly, immediately after the decapitation, whole blood was collected into cold tube containing inhibitor cocktail (5 mM ethylendiamintetraacetic acid (EDTA), 1.25 mM 1,10-phenanthroline, 20 μM enalapril maleate, 10 μM pepstatin A), kidneys and heart were removed, surface dried using gauze swabs, weighed and 0.5 g of the tissue was homogenized in 3 mL precooled methanol and centrifuged at 4°C and 3,000 g for 10 min. 1 mL of plasma for ANG II determination and 2 mL of plasma for ANG 1-7 measurement were precipitated with 4 mL of ethanol and again centrifuged. Supernatants were evaporated using Savant SpeedVac vacuum centrifuge. In addition, for ANG II determination only kidney samples were purified by solid-phase extraction (SPE); for ANG 1–7 both plasma and kidney samples were purified by SPE. Eluates were evaporated. Dried plasma and kidney samples were stored at −20°C or lower until assayed.

### 2.4 Experimental design–series 3

The aim of this series was to find out the degree of cardiac tolerance to ischemia/reperfusion (I/R) injury. The following groups were studied:

1. Noninduced (NI) rats/untreated (*n* = 15)
2. Noninduced (NI) rats/losartan (*n* = 11)
3. 13 days I3C-induced/untreated (*n* = 11)
4. 13 days I3C-induced/losartan (*n* = 12)

At the end of experimental protocol, rats were subjected to regional myocardial I/R as described previously, using an open-chest model (Neckár et al., 2012; Alánová et al., 2015; Hrdlička et al., 2019). Animals were anesthetized with pentobarbital sodium (60 mg/kg body weight i. p.). A heparinized cannula was placed in the left carotid artery for BP monitoring with a pressure transducer (Gould P23Gb; Gould Instruments Systems, Valley View, Ohio, United States). Intubated rats were ventilated with room air at 68 strokes/min (Ugo Basile, Italy; tidal volume of 1.2 mL/100 g body weight), and the rectal temperature was maintained between 36.5°C and 37.5°C using a heating pad throughout the experiment. Left thoracotomy was performed, and a silk suture 6-0 (Chirmax, Czech Republic) was placed loosely around the left anterior descending coronary artery 1–2 mm distal to its origin. After 10 min stabilization, regional ischemia was induced by tightening of the suture threaded through a polyethylene tube. Characteristic changes in myocardial color and the incidence of ischemic arrhythmias verified the complete coronary artery occlusion. After 20 min of regional ischemia, reperfusion of previously ischemic tissue continued for 3 h. At the end of reperfusion, the heart was excised and washed with saline via the aorta. The infarct size and the area at risk were determined by staining with potassium permanganate and 2,3,5-triphenyltetrazolium chloride (Sigma Aldrich, Prague, Czech Republic), respectively. The infarct size was normalized to the area at risk. The weight of the left ventricle (LV) was determined, and the area at risk was normalized to the LV. This approach has been validated in many experimental studies and is currently accepted as the “gold standard” for measuring myocardial infarct size in small animals (Sanada et al., 2011). The incidence and severity of ventricular arrhythmias during the 20 min ischemia and first 3 min of reperfusion were assessed according to the Lambeth Conventions (Walker et al., 1988). Premature ventricular complexes (PVCs) occurring as singles, salvos (2 or 3 PVCs), or tachycardia (VT, a run of 4 or more consecutive PVCs) were counted separately. The incidence and duration of life-threatening ventricular tachyarrhythmias, i.e., VT and fibrillation (VF), were also determined. VF lasting more than 2 min was considered as sustained (sVF). Rat hearts exhibiting VFs were excluded from further evaluation, such hearts are exposed to a tremendous ischemic insult that further alters the infarct size. However, the data were used for analysis of incidence and severity of arrhythmias.

### 2.5 Statistical analysis

Data are expressed as means ± SEM. Graph-Pad Prism software (Graph Pad Software, San Diego, California, United States) was used for statistical analysis. Multiple-group comparisons were performed by regular two-way analysis of variance, followed by Tukey´s multiple comparisons test as appropriate. The incidence of tachycardia and fibrillation was examined by Fischer’s exact test. One-way ANOVA with Tukey´s test was performed in normally distributed variables. Differences in arrhythmias among the groups were compared by the Kruskal–Wallis nonparametric test. Values exceeding the 95% probability limits (*p* < 0.05) were considered statistically significant. The data that were not normally distributed (arrhythmias) were expressed as median_interquartile range.

## 3 Results

### 3.1 Series 1: echocardiography and pressure-volume analysis

**FIGURE 1:**
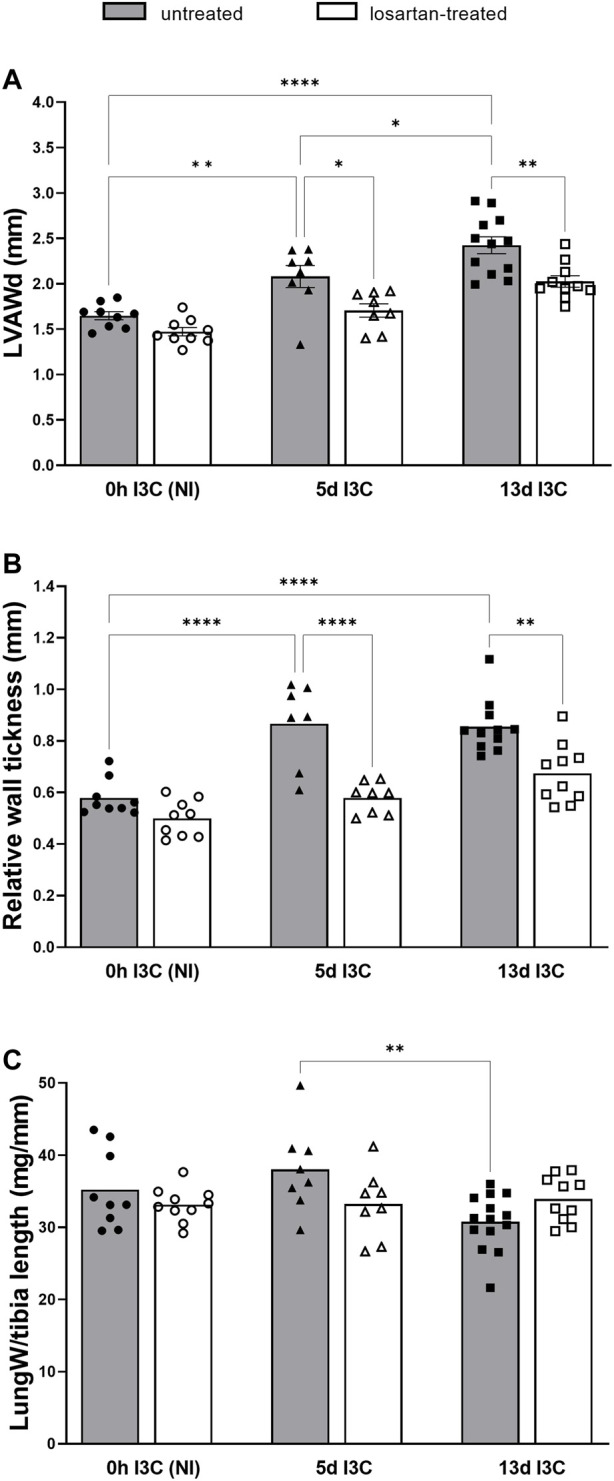
Left ventricular anterior wall tickness (LVAWd) **(A)**, relative wall tickness (RWT) **(B)** and lung weight (LungW) to tibia length ratio **(C)** in untreated and losartan-treated noninduced (NI) and I3C-induced Cyp1a1-Ren-2 transgenic rats. Values are expressed as means ± SEM. *p < 0.05, **p < 0.01, ****p < 0.0001.

**FIGURE 2:**
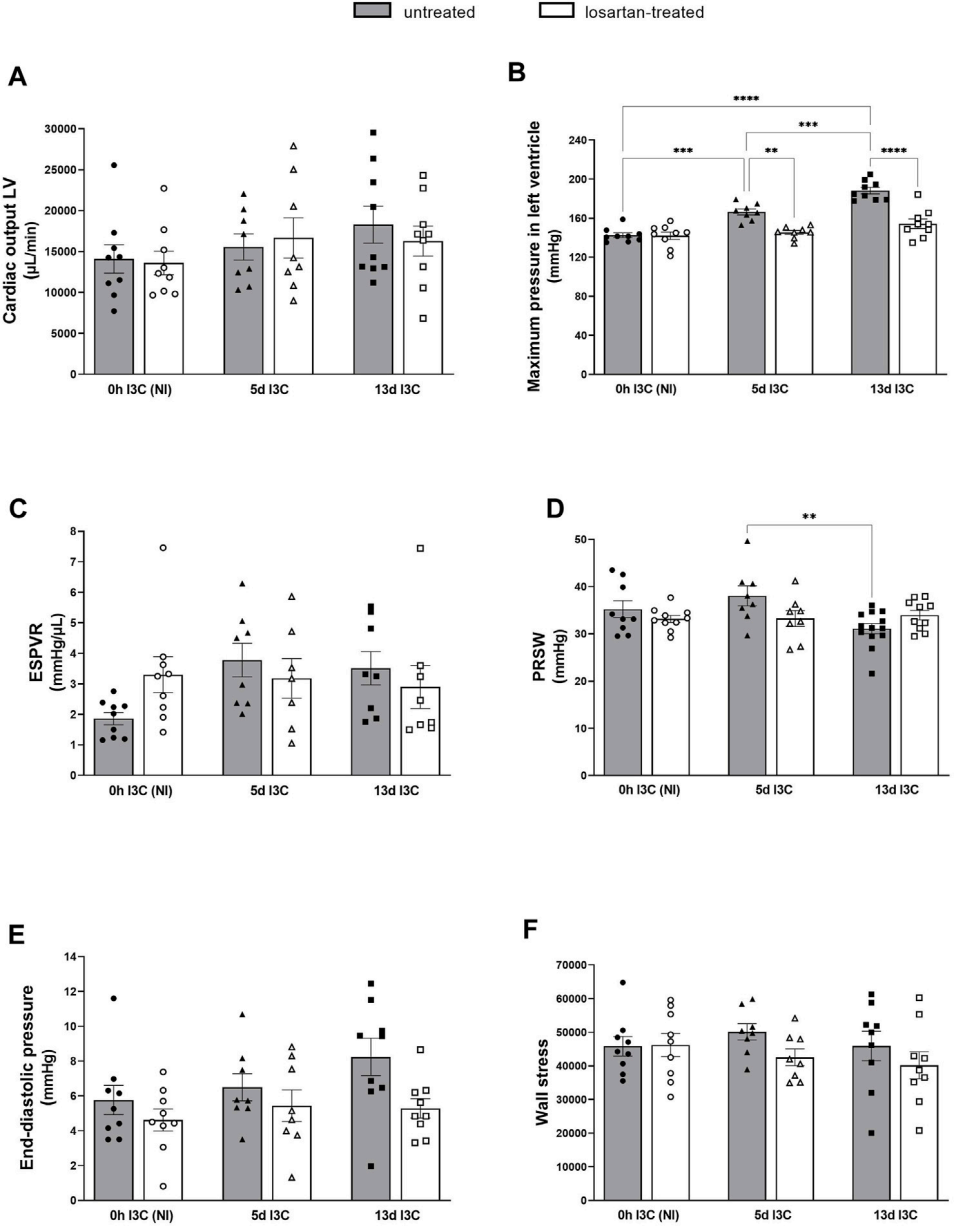
Cardiac output of left ventricle **(A)**, maximum pressure in left ventricle **(B)**, end-systolic pressure volume relationship (ESPVR) **(C)**, preload recruitable stroke work (PRSW) **(D)**, end-diastolic pressure **(E)** and wall stress **(F)** in untreated and losartan-treated noninduced (NI) and I3C-induced Cyp1a1-Ren-2 transgenic rats. Values are expressed as means ± SEM. **p < 0.01, ***p < 0.001, ****p < 0.0001.

Cardiac function was examined by non-invasive echocardiography and invasive pressure-volume analysis (Figures 1, 2).

I3C-induced rats developed LV hypertrophy, showed by left ventricular anterior wall thickness (LVAWd) and by relative wall thickness (RWT, anterior plus posterior wall thickness divided by the left ventricular diastolic diameter) in comparison to noninduced (NI) rats (Figures 1A, B). The severity of concentric LV hypertrophy was reduced by losartan treatment. 13 days induction led to a significant decrease of the lung weight to tibia length ratio in comparison to 5 days induction (Figure 1C). Losartan treatment abolished this difference. No significant changes in RV diameters and function were observed.

There were no significant differences in cardiac output, end-systolic pressure volume relationship (ESPVR), end-diastolic pressure and wall stress (maximum LV pressure x maximum LV volume)/LV weight) between I3C-induced and NI rats, without any marked influence of the treatment (Figures 2A, C, E, F). Untreated rats displayed increased maximum pressure in LV 5 days (5 days) and 13 days (13 days) after I3C-induction in comparison to NI rats, which was reduced by losartan treatment (145.3 ± 2.1 and 154.4 ± 4.9 vs. 166.5 ± 3.1 and 188.2 ± 3.3 mmHg, respectively) (Figure 2B). The preload recruitable stroke work (PRSW) is an index of myocardial contractility, that is, insensitive to preload and afterload. 13 days induction resulted in a substantial decrease of PRSW in comparison to 5 days induction, indicating an impaired myocardial function; losartan treatment did not affect it (Figure 2D). On the other hand, there were only nonsignificant trends toward worsening of the other systolic and diastolic parameters (e.g., end-diastolic pressure, *p* = 0.6690), showing that the myocardium was still in a compensated phase of its dysfunction, which is also documented by missing lung congestion (Figure 1C). Representative steady-state pressure-volume loops of all experimental groups are shown in Supplementary Figure S1.

### 3.2 Series 2: renin-angiotensin system

**FIGURE 3:**
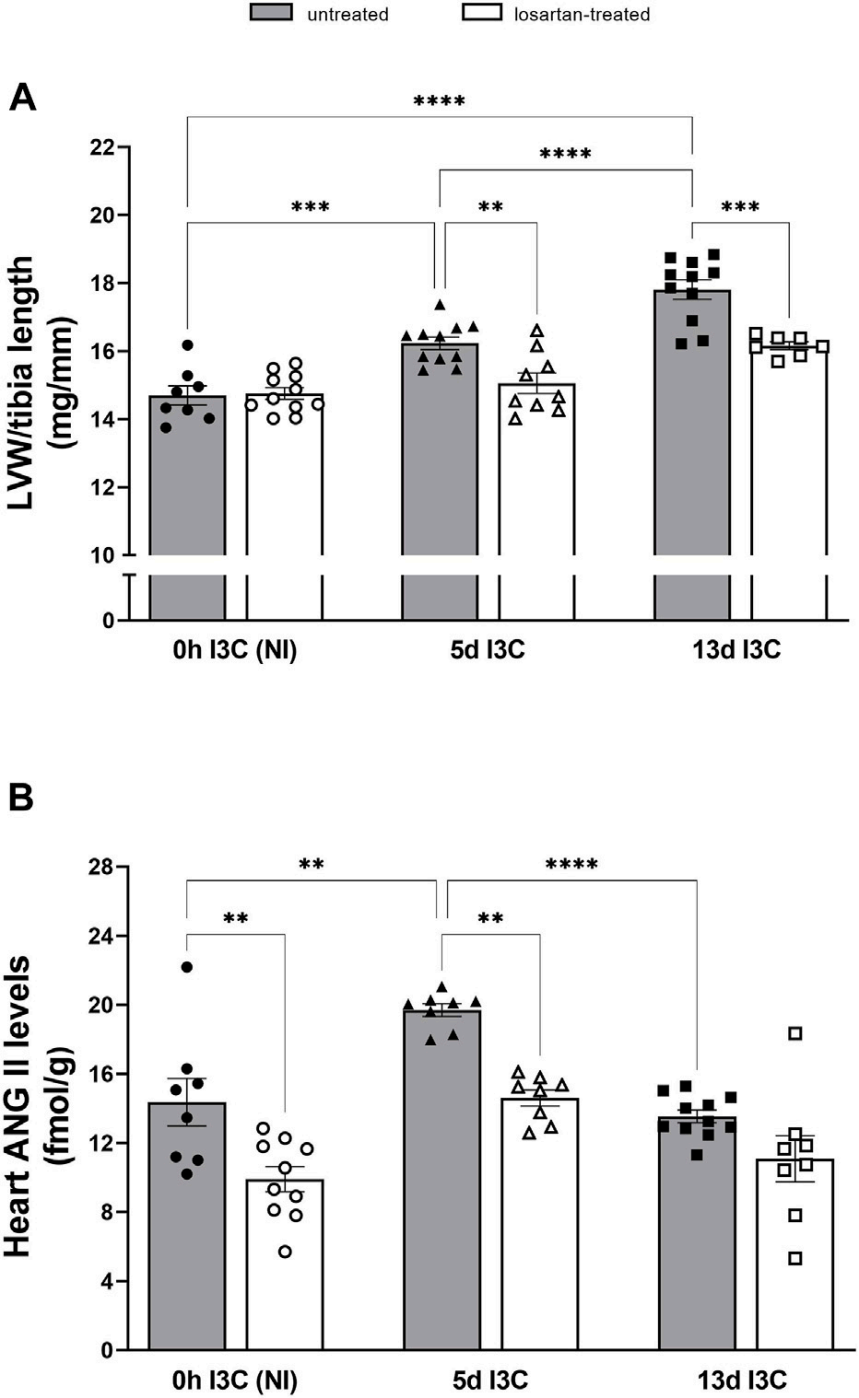
The ratio of left ventricular weight (LVW) to tibia length **(A)** and heart angiotensin II (ANG II) levels **(B)** in untreated and losartan-treated noninduced (NI) and I3C-induced Cyp1a1-Ren-2 transgenic rats. Values are expressed as means ± SEM. **p < 0.01, ***p < 0.001, ****p < 0.0001.

The degree of cardiac hypertrophy was assessed as the ratio of left ventricular weight (LVW) to tibia length. Untreated rats developed marked cardiac hypertrophy after 5 and 13 days of I3C-induction compared to NI rats, which was reduced by losartan treatment (15.1 ± 0.3 and 16.2 ± 0.2 vs. 16.2 ± 0.1 and 17.8 ± 0.3 mg/mm, respectively) (Figure 3A). The increased heart ANG II levels in 5 days I3C-induced rats compared to NI rats (19.7 ± 0.4 vs. 14.4 ± 1.4 fmol/g), that were attenuated by losartan treatment, were reduced after 13 days I3C-induction back to the levels as observed in NI rats (Figure 3B).

**FIGURE 4:**
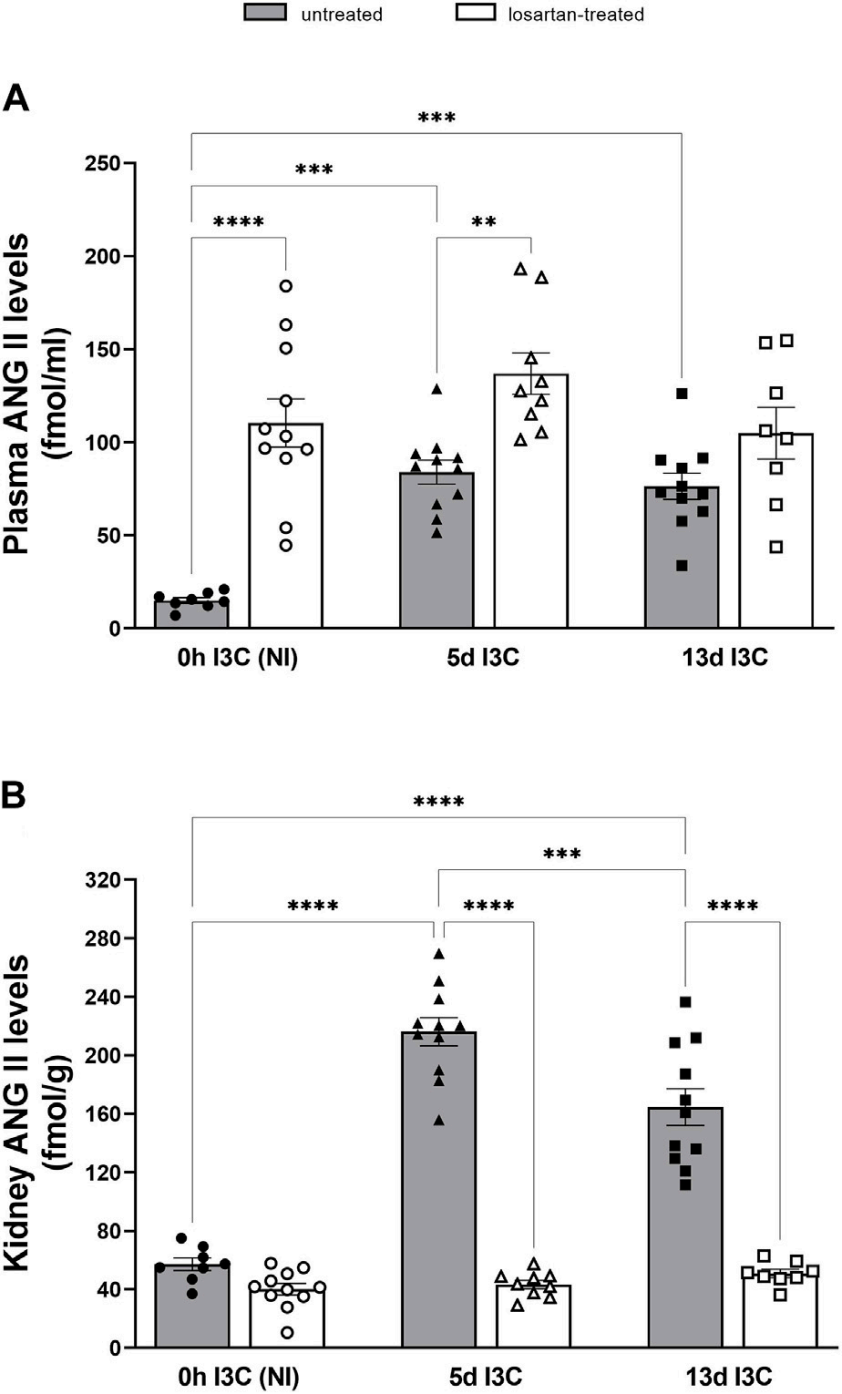
Plasma angiotensin II (ANG II) levels **(A)** and kidney ANG II levels **(B)** in untreated and losartan-treated noninduced (NI) and I3C-induced Cyp1a1-Ren-2 transgenic rats. Values are expressed as means ± SEM. **p < 0.01, ***p < 0.001, ****p < 0.0001.

Both 5 and 13 days I3C-induction resulted in a significant rise of plasma ANG II levels in comparison to NI rats (84 ± 6.4 and 76.4 ± 7.0 vs. 15.0 ± 1.5 fmol/mL) (Figure 4A). Losartan treatment significantly increased plasma concentrations of ANG II in NI and 5 days I3C-induced rats, with a nonsignificant trend toward an increase in 13 days I3C-induced rats (*p* = 0.3473). 5 days I3C-induction led to about 4-fold higher kidney ANG II levels in comparison to NI rats, which were significantly reduced after 13 days I3C-induction, but to levels statistically higher than NI rats (164.6 ± 12.6 vs. 216.1 ± 9.6 vs. 57.2 ± 4.2 fmol/g). These effects were reduced by losartan treatment to the level observed in NI rats (Figure 4B).

**FIGURE 5:**
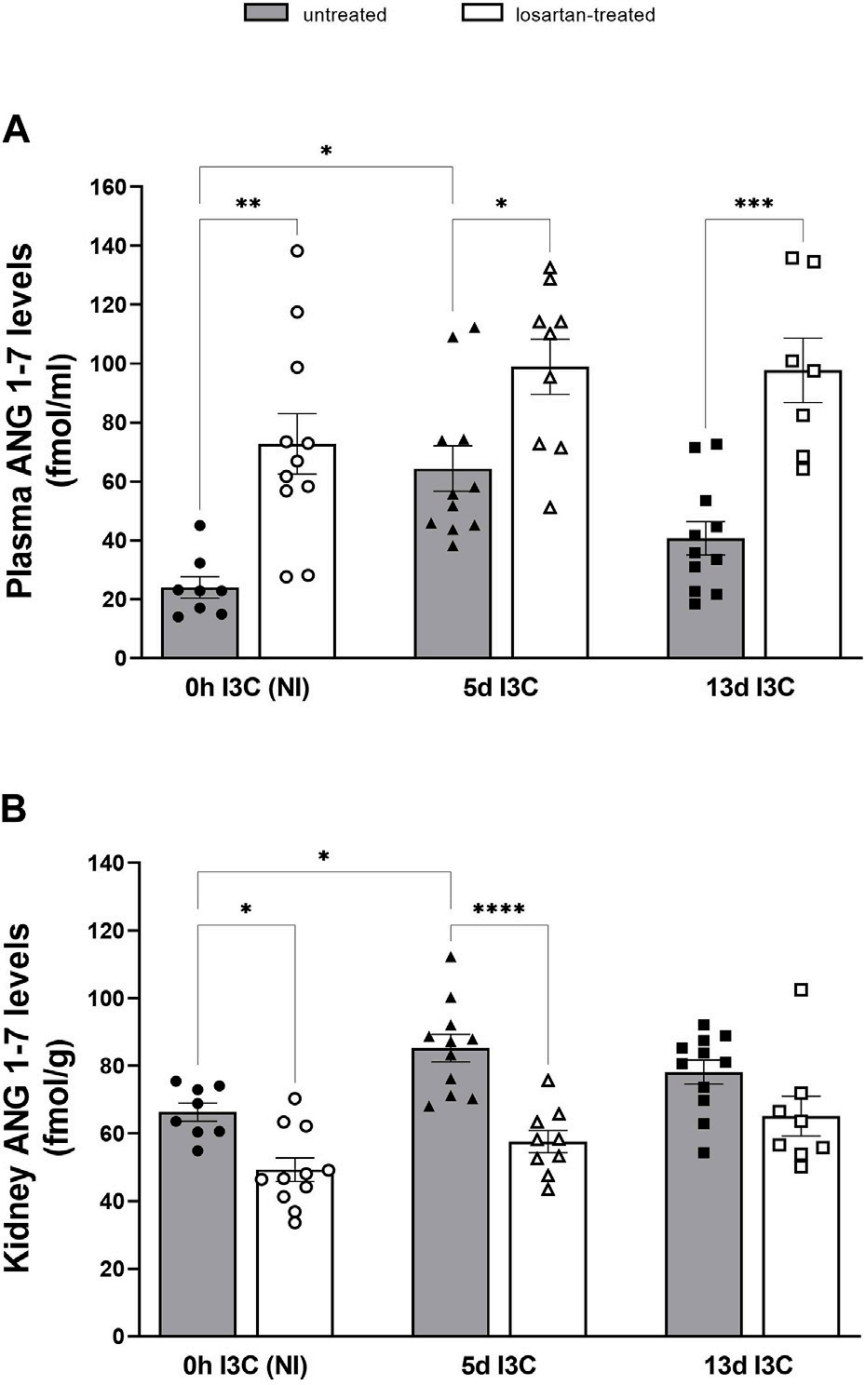
Plasma angiotensin 1–7 (ANG 1-7) levels **(A)** and kidney ANG 1-7 levels **(B)** in untreated and losartan-treated noninduced (NI) and I3C-induced Cyp1a1-Ren-2 transgenic rats. Values are expressed as means ± SEM. *p < 0.05, **p < 0.01, ***p < 0.001, ****p < 0.0001.

As shown in Figure 5A, plasma angiotensin 1–7 (ANG 1–7) levels in 5 days I3C-induced rats were significantly higher in comparison to NI (64.4. ± 7.7 vs. 24.0 ± 3.6 fmol/mL). Losartan treatment significantly increased plasma ANG 1–7 levels in NI and induced rats throughout the experiment. We observed significant difference in kidney ANG 1–7 levels between NI and 5 days I3C-induced rats (Figure 5B). Longer I3C-induction did not lead to further increase. Losartan treatment significantly decreased renal concentration of ANG 1-7 in 5 days I3C-induced rats (57.6 ± 3.3 vs. 85.2 ± 4.1 fmol/g).

**FIGURE 6:**
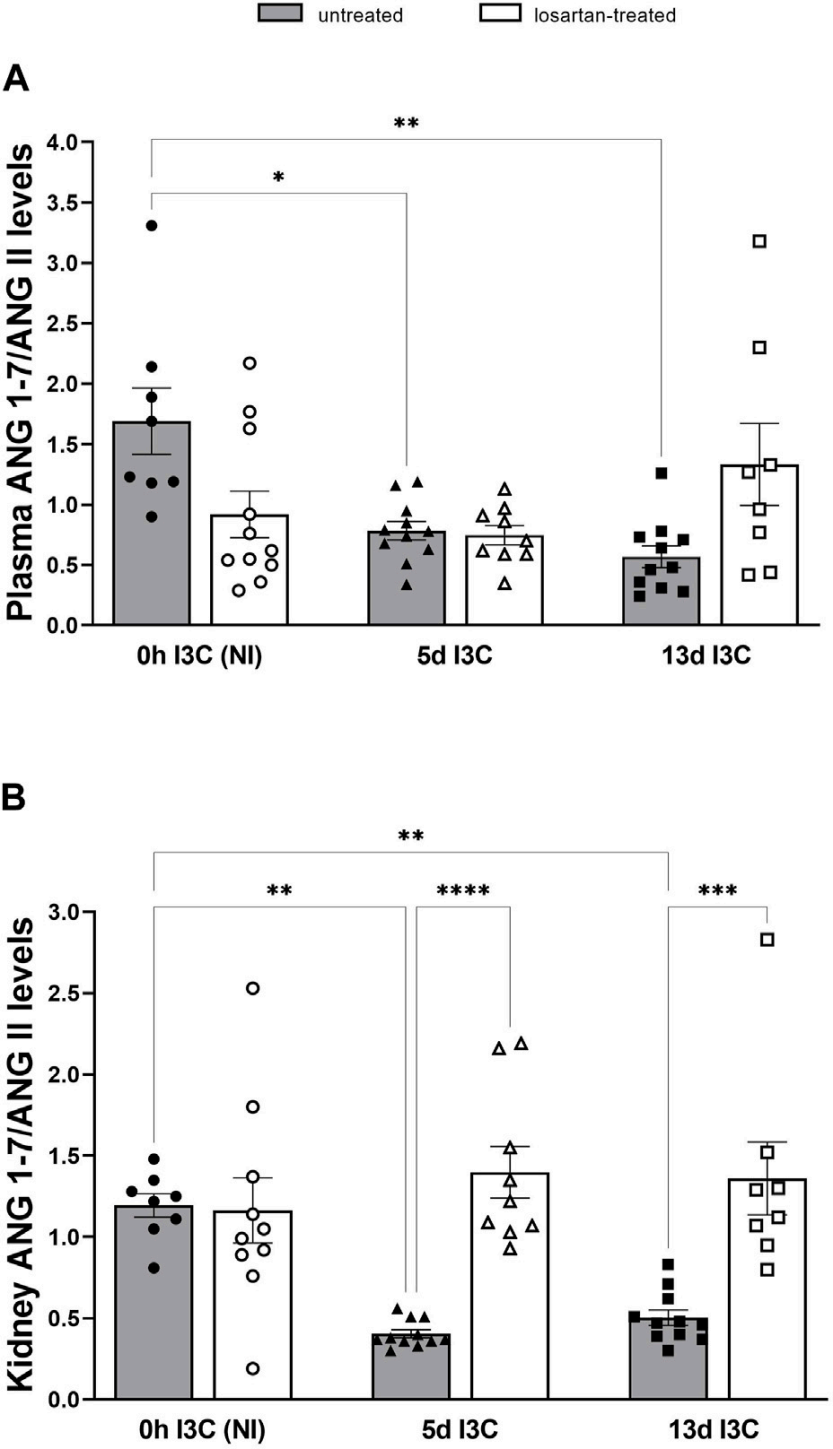
Plasma angiotensin 1–7 (ANG 1-7)/angiotensin II (ANG II) levels **(A)** and kidney ANG 1-7/ANG II levels **(B)** in untreated and losartan-treated noninduced (NI) and I3C-induced Cyp1a1-Ren-2 transgenic rats. Values are expressed as means ± SEM. *p < 0.05, **p < 0.01, ***p < 0.001, ****p < 0.0001.

Both plasma and kidney ANG 1-7/ANG II ratios were suppressed after 5 and 13 days of I3C-induction compared to NI rats (Figures 6A, B). Losartan treatment did not affect plasma ANG 1-7/ANG II ratio, but it restored values of kidney ANG 1-7/ANG II ratio to levels comparable to those of treated NI rats.

### 3.3 Series 3: heart studies–Ischemia/reperfusion injury

Temporary or permanent left coronary artery (LCA) ligation is the most widely used model of heart failure in rats.

**FIGURE 7:**
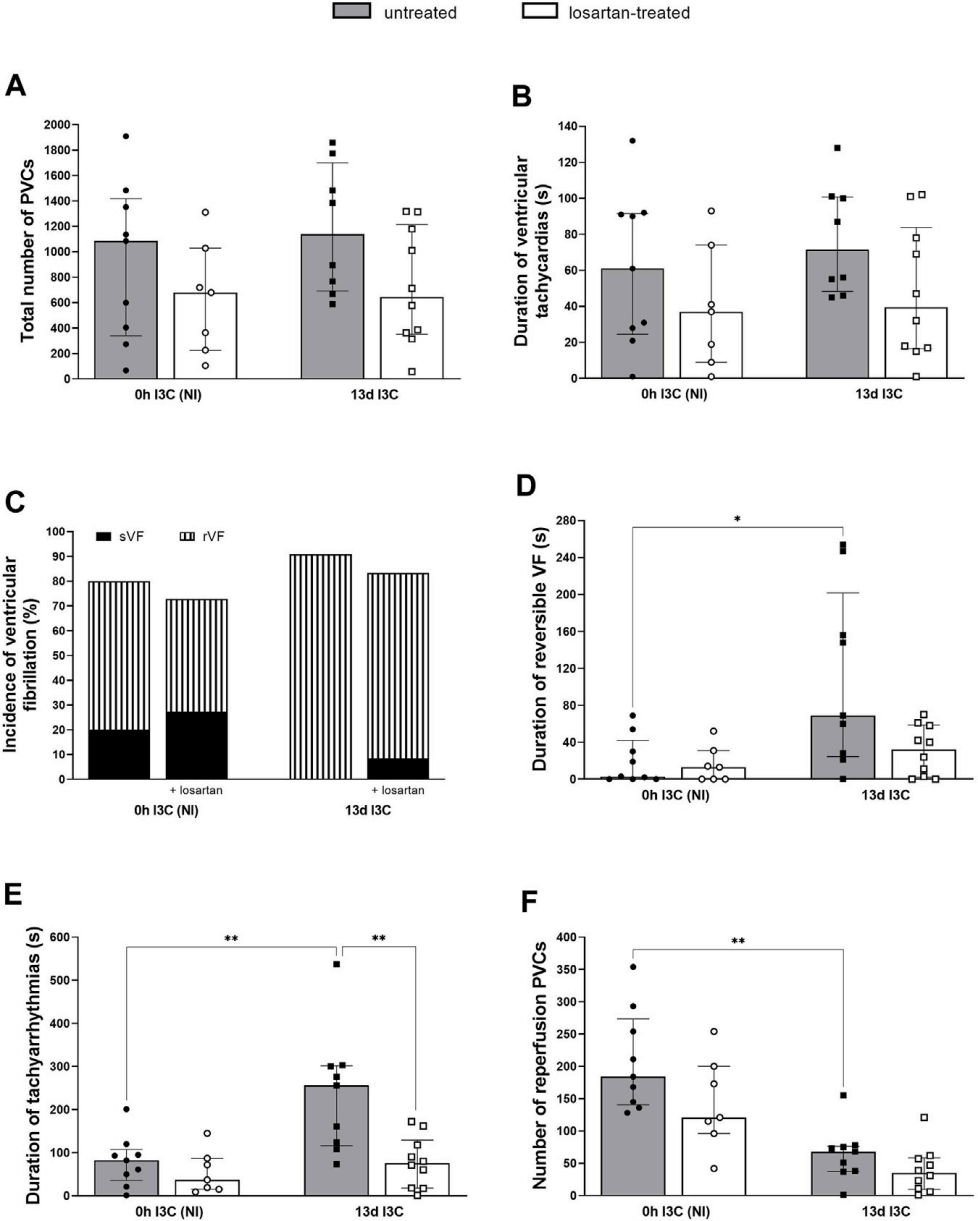
Total number of premature ventricular complexes (PVCs) **(A)**, duration of ventricular tachyarrhythmias **(B)**, the incidence of ventricular fibrillation, both reversible and sustained **(C)**, duration of reversible ventricular fibrillation (VF) **(D)** and tachyarrhythmias **(E)** and number of reperfusion premature ventricular complexes (PVCs) **(F)** in untreated and losartan-treated noninduced (NI) and I3C-induced Cyp1a1-Ren-2 transgenic rats. Values are expressed as means ± SEM or as median with interquartile range. *p < 0.05, **p < 0.01.

Untreated NI and 13 days I3C-induced groups did not differ in the number of ischemic PVCs, the duration of ventricular tachycardias and the incidence of all ventricular fibrillations (VF) (Figures 7A–C). There was a trend, but nonsignificant, toward a reduction in the number of ischemic PVCs and the duration of ventricular tachycardias in losartan-treated rats compared to untreated groups. Still, it did not reach statistical significance due to high variability. The time of reversible VF (rVF) and tachyarrhythmias was significantly longer in 13 days I3C-induced rats compared to NI rats (Figures 7D, E). Losartan treatment shortened the duration of tachyarrhythmias in 13 days I3C-induced group. The number of reperfusion PVCs was significantly lower in 13 days I3C-induced groups compared to NI groups (Figure 7F).

**FIGURE 8:**
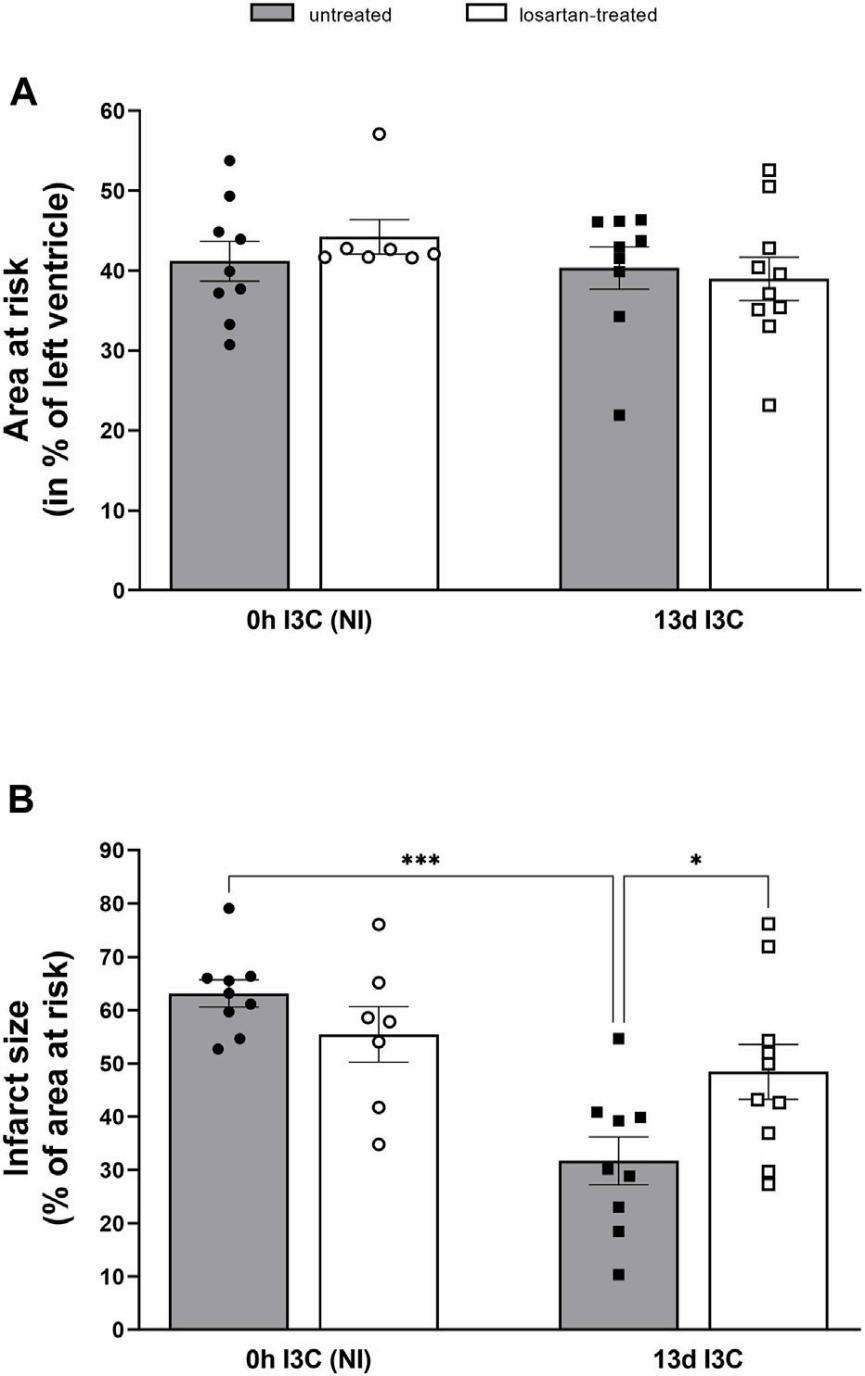
Myocardial area at risk normalized to the size of the left heart ventricle **(A)** and infarct size presented as a percentage of the area at risk **(B)** in untreated and losartan-treated noninduced (NI) and I3C-induced Cyp1a1-Ren-2 transgenic rats. Values are expressed as means ± SEM. *p < 0.05, ***p < 0.001.

The mean area at risk (normalized to the size of the LV) did not significantly differ among groups, and it ranged between 39%–43% (Figure 8A). The myocardial infarct size (IS; normalized to the area at risk) was significantly reduced in 13 days I3C-induced rats in comparison to NI group (31.7% ± 4.5% vs. 63.1% ± 2.6%) (Figure 8B). Losartan treatment significantly increased IS in I3C-induced rats to 48.4% ± 5.1%. Representative histological images of myocardial infarction of all experimental groups are shown in Supplementary Figure S2.

**FIGURE 9:**
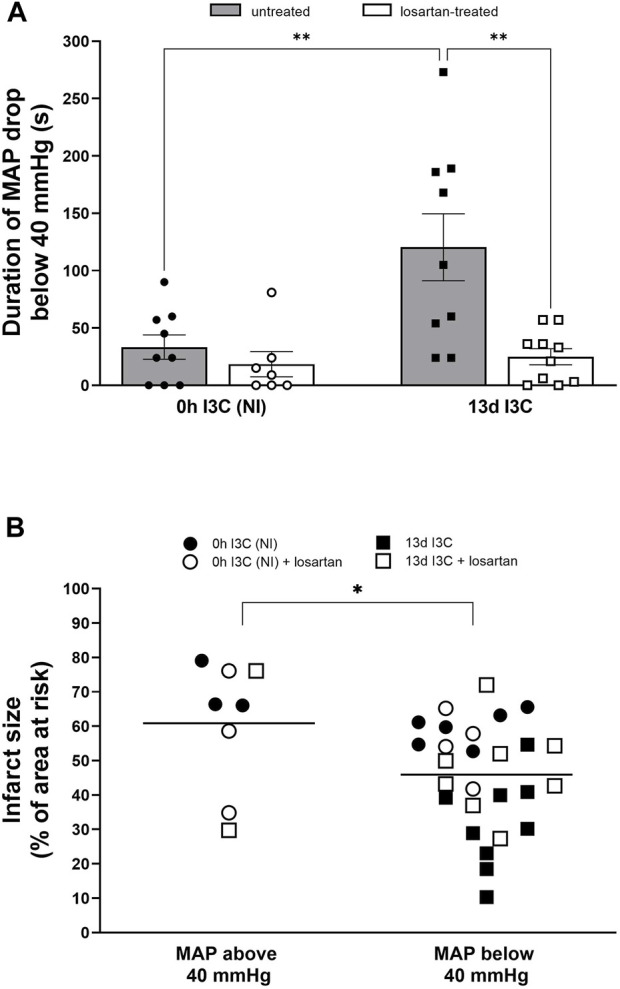
Duration of mean arterial pressure (MAP) drop below 40 mmHg **(A)** and infarct size (presented as a percentage of the area at risk) with or without MAP drops below 40 mmHg **(B)** in untreated and losartan-treated noninduced (NI) and I3C-induced Cyp1a1-Ren-2 transgenic rats. Values are expressed as means ± SEM **(A)** and grand means **(B)**. *p < 0.05, **p < 0.01.

Severe ischemic arrhythmias can cause a substantial drop in blood pressure leading to insufficient perfusion of organs and tissues (Neckář et al., 2021). Animal studies suggested that mean arterial pressure (MAP) below 40 mmHg is a value of MAP close to the critical closing pressure for the whole circulation (Magder, 2018). During ischemia, MAP drop below 40 mmHg was markedly longer in I3C-induced rats due to longer ventricular tachyarrhythmias compared to NI rats and was shortened by losartan treatment (Figure 9A). MAP dropped below 40 mmHg in all untreated and in 8 out of 10 of I3C-induced rats; in these rats, the infarct size was significantly reduced (Figure 9B). These findings demonstrate myocardial protection against infarction, that is, likely mediated by protective substances released from body organs and tissues made hypoxic due to exaggerated ischemic tachyarrhythmias as we demonstrated recently (Neckar et al., 2021).

## 4 Discussion

This study tested our hypothesis, that in the late phase of hypertension with already developed signs of end-organ damage, inappropriate activation of RAS impairs cardiac tolerance to I/R injury. As the optimal model for this study, we chose transgenic rats with inducible hypertension, in which an inappropriately activated vasoconstrictive axis of the RAS became a major contributor to the development of ANG II-dependent hypertension and substantially LVH.

The most important finding of the present study is that the infarct size (IS) was significantly reduced (by 50%) in 13 days I3C-induced hypertensive rats with marked cardiac hypertrophy compared to NI normotensive rats. That indicates improved cardiac tolerance to I/R injury and, at the same time, shows that our hypothesis was incorrect. Increased myocardial resistance to I/R injury was accompanied by a longer duration of rVF and tachyarrhythmias but also by a significantly reduced number of ischemic sVF and reperfusion PVCs. That is, in accordance with our previous study in transgenic rats with inducible hypertension (Husková et al., 2021) and with the observations in a model of human renovascular hypertension (Alánová et al., 2015) or in TGR (Neckár et al., 2012), where improved cardiac tolerance to I/R injury was also confirmed. Moreover, all of I3C-induced rats reached the critical whole-circulatory closing pressure of 40 mmHg during ischemia and remained there for longer than NI rats. Prolonged ischemic tachyarrhythmias resulted in transient blood pressure decreases associated with insufficient organ and tissue perfusion, which was accompanied by an IS-reducing effect. The same effect of IS reduction was observed in SHR-CRP rat strain (Magder, 2018; Neckář et al., 2021).

To better understand the structure and function of the heart and the underlying mechanism(s), we decided to perform non-invasive echocardiography and invasive pressure-volume analysis of the left ventricle. We confirmed concentring cardiac hypertrophy, increased maximum wall thickness, and decreased inner left ventricle diameter in untreated I3C-induced rats. We observed indications of myocardial dysfunction, mainly in reduced PRSW, but only nonsignificant trends in worsening of some other parameters, showing that the myocardium is in a compensated phase. It is evident that high afterload after a long I3C-induction is not beneficial for the left ventricle, and even if it is not yet heart failure, we can conclude that it is the beginning of the preclinical phase, without clinical signs of heart failure (e.g., lung congestion).

Elevated plasma ANG 1-7/ANG II ratio was identified as an independent and incremental predictor of beneficial outcomes, higher survival rate, and decreased hospitalization duration in patients with heart failure (Wang et al., 2020). The main effector of vasodilatory axis of the RAS, ANG 1–7 and ACE2, are important predictive factors for the severity of heart failure and myocardial remodeling of HF with preserved ejection fraction with hypertension (Yu et al., 2018). Several studies have shown that ANG 1–7 is cardioprotective following I/R injury and reduces cardiac hypertrophy (Reudelhuber, 2006; Tallant et al., 2006; Mercure et al., 2008; Stewart et al., 2008; Souza et al., 2013). We showed markedly elevated plasma and tissue ANG II and ANG 1–7 levels after 5 days of I3C-induction, in the early stage of hypertension. Longer I3C-induction did not lead to a further increase in angiotensins’ levels; on contrary, it resulted in a significant decrease in heart and kidney ANG II levels. From the calculation of plasma and kidney ANG 1–7/ANG II ratio, it is clear that the vasodilatory axis of the RAS is suppressed after both 5 and 13 days of I3C-induction. In our previous study, we found that kidney ANG 1–7 levels were twice as high as the levels of ANG II after 12-h induction and the longer 5 days of I3C-induction led to a faster increase in the ANG II levels (Husková et al., 2021). In this study, we confirmed the activation of both axes of the RAS during the development of hypertension. It is apparent that it depends on the stage of hypertension, in the initial stage, the vasodilatory axis of the RAS prevails, and with the development of hypertension, the vasoconstrictive axis of the RAS becomes stronger.

ANG II type 1 receptor blockers (ARBs) have been effectively used in hypertension and HF. They are essential antihypertensive agents suitable for both monotherapy and combination treatment. ARBs have comparable blood pressure-lowering effects to other major antihypertensive drugs, including ACE inhibitors and calcium channel blockers. They downregulate the ACE/ANG II/AT1 axis and upregulate the ACE2/ANG 1–7/Mas axis. In our study, we used losartan to study a possible cardioprotective mechanism. We confirmed the apparent positive effect of losartan treatment on maximum pressure in LV and cardiac hypertrophy. The RAS activity inhibition by specific AT1 receptor antagonist resulted in a marked elevation of plasma ANG II levels. This results from the interruption of a strong negative feedback loop where AT1 activation suppresses renin secretion and thereby reduces plasma ANG II levels (Castrop et al., 2010; Kurtz, 2012). Chronic blockade of the AT1 receptor also increased plasma ANG 1–7, which mediates mechanisms counterregulatory to AT1 signaling. AT1 receptor blockade normalized duration of tachyarrhythmias and infarct size in 13 days I3C-induced rats to levels comparable to NI rats. As the reduced infarct size was abolished by losartan treatment, this points to an important role of AT1 receptors and their main activator ANG II in cardiac tolerance to I/R injury during the late phase of hypertension. Unfortunately, we did not confirm increased local production of ANG II in the heart of 13 days I3C-induced rats. AT1 receptor expressed in the cardiovascular system has been shown to activate a variety of intracellular protein kinases that stimulate NADPH oxidase, ROS generation and protein synthesis, causing hypertrophy, hyperplasia and migration of VSMCs, cardiac hypertrophy and renal deterioration (Kawai et al., 2017). Our current study showed that losartan, in the late phase of hypertension, does not have as many positive cardioprotective and antiarrhythmic effects as it did in the early phase of hypertension (Husková et al., 2021).

There is increasing evidence that some animal models of hypertension may also activate mechanisms that temporarily increase cardiac protection against acute ischemia/reperfusion injury. Several studies (including ours) using different genetic models of hypertension have shown that myocardial infarction can be significantly reduced in hypertensive animals compared to normotensive controls (Mozaffari & Schaffer, 2003; Neckar et al., 2012; Alánová et al., 2015). As we have shown in this study, the decrease in perfusion pressure due to prolonged ischemic tachyarrhythmias can cause a reduction in myocardial infarction in 13 days I3C-induced hypertensive rats. The tachyarrhythmia-induced decrease in MAP could be responsible for the subsequent release of various substances into the blood from hypoxic organs and tissues during reperfusion, as we have published in SHR (Neckar et al., 2021). In this work, we have also shown that increased plasma levels of some metabolites (e.g., pyruvate, succinate, urate, or ketone bodies) not only reflect organ and tissue hypoxia but have been identified as possible cardioprotective molecules (Teng et al., 2002; Snorek et al., 2012; Mallet et al., 2018; Yu et al., 2018; Zhang et al., 2018). However, further studies will be needed to clarify the contribution of these (and others) metabolites to cardioprotective signaling induced by excessive ischemic arrhythmias in both SHR and 13 days I3C-induced hypertensive rat strains. Overall, this novel form of cardioprotection is initiated by the heart itself (i.e., “self-conditioning”), without any external intervention such as targeted brief occlusion of another organ to activate various forms of remote conditioning (Schmidt et al., 2007; Kleinbongard et al., 2018). We hypothesize that “self-conditioning” represents a general cardioprotective phenomenon that may paradoxically increase cardiac ischemia tolerance in rats with various comorbidities.

In summary, we confirmed the different activity of the RAS axes during the development of ANG II-dependent hypertension, the influence of the RAS depends on the balance between them. In the initial stage of hypertension, the vasodilatory axis of the RAS prevails, and with the development of hypertension the vasoconstrictive axis of the RAS becomes stronger. The infarct size was significantly reduced (by 50%) in 13 days I3C-induced hypertensive hypertrophied rats, this indicates improved cardiac tolerance to I/R injury. The infarct size reduction was reversed by losartan treatment. In the late phase of hypertension there are indications of a failing heart, mainly in reduced PRSW, but only nonsignificant trends in worsening of some other parameters, showing that the myocardium is in a compensated phase. We observed a clear effect of AT1 receptor blockade on maximum pressure in left ventricle, cardiac hypertrophy and ANG II levels.

## Figures and Tables

**Table:** 

**ACE**	angiotensin-converting enzyme
**ACE2**	angiotensin-converting enzyme 2
**ANG 1-7**	angiotensin 1-7
**ANG II**	angitensin II
**ARBs**	AT1 receptor blockers
**AT1**	ANG II receptor type 1
**BP**	blood pressure
**BW**	body weight
**EDTA**	ethylendiamintetraacetic acid
**ECHO**	echocardiography
**EDPVR**	end-diastolic pressure volume relationship
**ESPVR**	end-systolic pressure volume relationship
**HBP**	high blood pressure
**HF**	heart failure
**I3C**	indol-3-carbinol
**IHD**	ischemic heart disease
**I/R**	ischemia/reperfusion
**IS**	infarct size
**LCA**	left coronary artery
**LV**	left ventricle
**LVAWd**	left ventricular anterior wall thickness
**LVH**	left ventricular hypertrophy
**LVW**	left ventricular weight
**MAP**	mean arterial pressure
**NADPH**	Nicotinamide adenine dinucleotide phosphate
**Mas**	ANG 1-7 receptor
**NI**	noninduced
**PRSW**	preload recruitable stroke work
**P-V analysis**	presuure-volume analysis
**PVCs**	premature ventricular complexes
**RAS**	renin-angiotensin system
**RIA**	radioimmunoassay
**ROS**	radical oxygen species
**RWT**	relative wall tickness
**rVF**	reversible ventricular fibrillation
**SPE**	solid-phase extraction
**sVF**	sustained ventricular fibrillation
**TGR**	mouse Ren-2 renin transgenic rats
**VSMCs**	vascular smooth muscle cells
**VT**	ventricular tachycardia

## Data Availability

The original contributions presented in the study are included in the article/Supplementary Material, further inquiries can be directed to the corresponding author.
