# Supplementary material for: Inappropriate activation of the renin-angiotensin system improves cardiac tolerance to ischemia/reperfusion injury in rats with late angiotensin II-dependent hypertension
Source: Front Physiol. 2023 Jun 14;14:1151308. doi: 10.3389/fphys.2023.1151308 (PMC10301744; doi:10.3389/fphys.2023.1151308)
Supplement: Supplementary file 1 [file DataSheet1.docx]

Supplementary Material

Inappropriate Activation of the Renin-Angiotensin System Improves Cardiac Tolerance to Ischemia/Reperfusion Injury in Rats with Late Angiotensin II-dependent Hypertension.

**Zuzana Husková^*^, Soňa Kikerlová, Matúš Miklovič, Petr Kala, František Papoušek, Jan Neckář**

*** Correspondence:** Zuzana Husková - [zuhs@ikem.cz](mailto:zuhs@ikem.cz)

# Supplementary figures


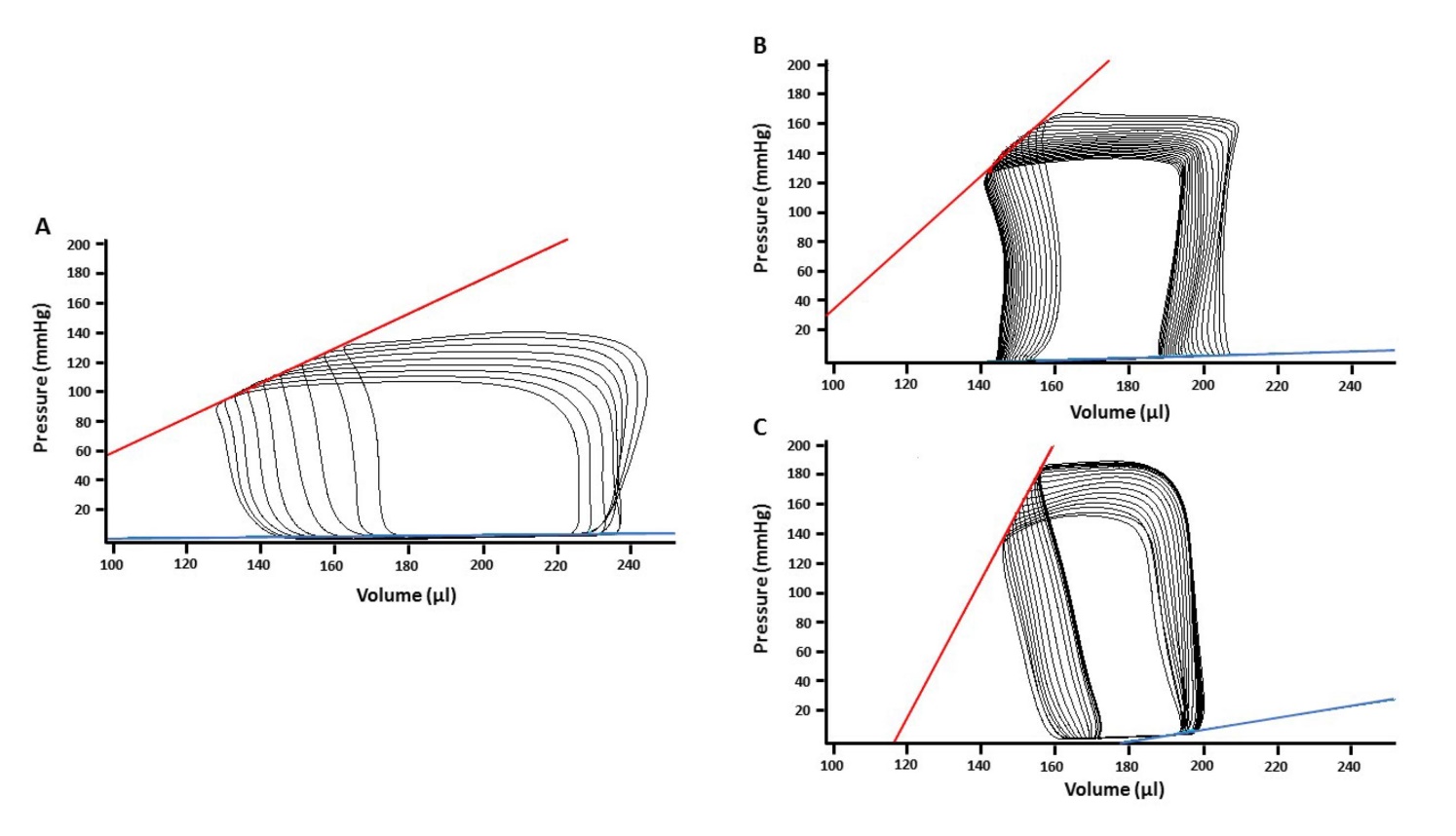


**Supplementary Figure S1.** Representative steady-state pressure-volume loops of control noninduced **(S1A)**, 5d **(S1B)** and 13d **(S1C)** I3C-induced rats. The slope of the end-systolic pressure-volume relationship (ESPVR; red line) was steeper and shifted to the left in I3C-induced rats compared with NI rats, indicating increased contractility. A steeper slope of the end-diastolic pressure-volume relationship (EDPVR; blue line) indicates decreased ventricular compliance in I3C-induced rats. Pressure-volume area (PVA), representing total mechanical energy generated by LV contraction, was lower in I3C-induced rats than in NI rats.


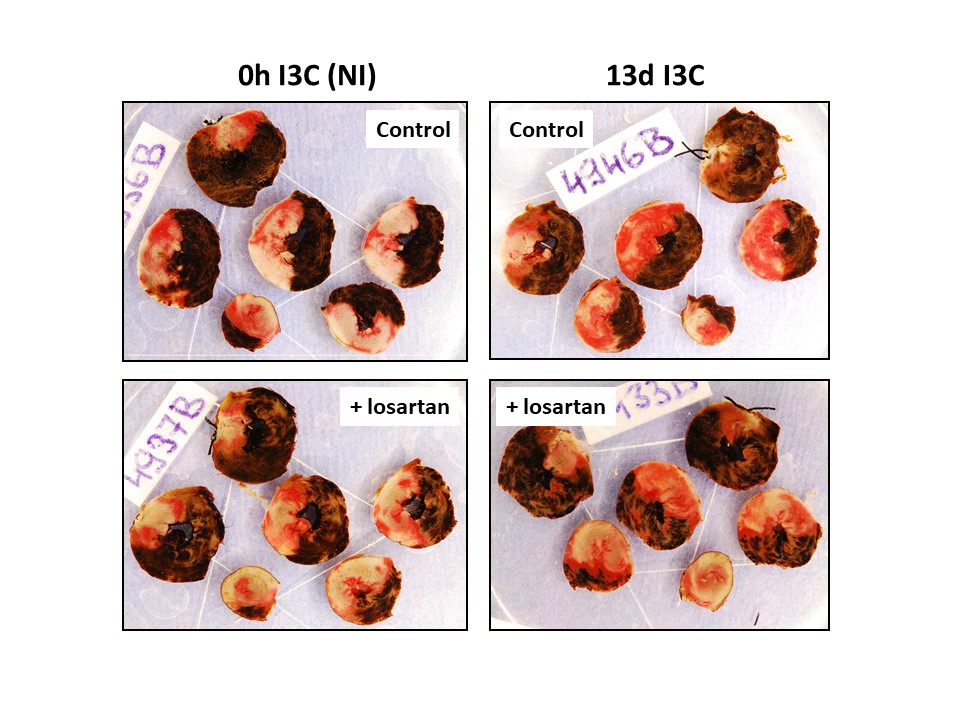
**Supplementary Figure S2.** Typical examples of myocardial infarction induced by 20min coronary artery occlusion and 3h reperfusion in untreated (control) and losartan-treated noninduced (NI) and 13d I3C-induced *Cyp1a1-Ren-2* transgenic rats. Brown color represents normally perfused tissue stained by potassium permanganate. Red area, tetrazolium positive, represents tissue surviving the occlusion, and pale area, tetrazolium negative, is infarcted tissue.
